# Factors predisposing to shared governance: a qualitative study

**DOI:** 10.1186/s12912-019-0334-2

**Published:** 2019-03-12

**Authors:** Foroozan Atashzadeh-Shoorideh, Mohammad-Mehdi Sadoughi, Maryam Sattarzadeh-Pashabeig, Alice Khachian, Mansoureh Zagheri-Tafreshi

**Affiliations:** 1 Department of Psychiatric Nursing, School of Nursing & Midwifery, Shahid Beheshti University of Medical Sciences, Vali-Asr Avenue, Cross of Vali-Asr Avenue and Hashemi Rafsanjani (Niyayesh) Highway, Opposite to Raja’ee Heart Hospital, Tehran, 1996835119 Iran; 2 Ophthalmology, School of Medicine, Shahid Beheshti University of Medical Sciences, Pasdaran St., Boostan-e-Nohom., Labbafi Nejad Medical Center, Tehran, Iran; 3 Student Research Committee, School of Nursing & Midwifery, Shahid Beheshti University of Medical Sciences, Vali-Asr Avenue, Cross of Vali-Asr Avenue and Hashemi Rafsanjani (Niyayesh) Highway, Opposite to Raja’ee Heart Hospital, Tehran, 1996835119 Iran; 4 School of Nursing and Midwifery, Department of Medical Surgical Nursing, Iran University of Medical Science, Vali-Asr Avenue, Rashid Yasemi St., Tehran, 1996713883 Iran; 5 Department of Nursing Management, School of Nursing & Midwifery, Shahid Beheshti University of Medical Sciences, Vali-Asr Avenue, Cross of Vali-Asr Avenue and Hashemi Rafsanjani (Niyayesh) Highway, Opposite to Raja’ee Heart Hospital, Tehran, 1996835119 Iran

**Keywords:** Shared governance, Education, Nursing, Schools, Qualitative research

## Abstract

**Background:**

The method of implementing shared governance varies among organizations. Identifying the predisposing factors can facilitate and precipitate its successful implementation and aid educational institutions in achieving their goals. This study determined the antecedents of shared governance in nursing schools.

**Methods:**

Eleven participants including faculty members of nursing schools, and managers of three major medical universities of Tehran were selected using purposive sampling method and underwent in-depth semi-structured interviews in this qualitative study. Conventional content analysis was used to analyze the data.

**Results:**

Data analysis led to the emergence of four categories including the participatory context of higher education institutions, infrastructural obligations, coordination with contemporary needs, and participation-oriented managers resulting in twelve subcategories.

**Conclusion:**

This study showed that managers can play a key role in the successful implementation of shared governance in the appropriate context of higher education institutions. Hence, the deliberate selection of managers who believe in managerial participation and their training are mandatory in nursing schools. The senior or higher level managers of educational institutions can empower themselves and their staff in participatory skills along with providing suitable resources of work serving as a suitable model of participation.

## Background

In recent years, the concept of institutional governance has been highlighted as one of the most important issues in many universities due to increasing complexity of duties and functions of higher education institutions and overwhelming pressures and expectations of the state, status of business, and other extra-organizational parameters. The educational institutes are asked to work more with smaller budgets while they are increasingly responsible for organizational and educational decision-makings. These issues along with other pressures have enhanced the importance of effective governance system in educational institutes [1]. Effective governance and promotion of the work environment are necessary and requires human resources. But in nursing schools, due to the progressive decline of faculty members and increasing number of students, the workload of faculty members is increasing, which demands an improvement in the quality of the work environment. However, nursing schools and most academic organizations act in a hierarchical and bureaucratic system of decision-making [2–4]. Moreover, faculty members generally deal with university students due to the nature of their job with a trivial role in policy- and decision-makings [3, 5].

Governance structures of educational institutions directly influence the faculty members and the staff and their occupational satisfaction [2]. It appears that shared governance has a decentered structure of power and decision-making [5, 6]. These factors have provided a position for faculty members parallel to that of managers to increase their participation to decision making processes and challenge the conventional governance [4]. Consequently, universities are required to direct their governance structure towards shared responsibilities [7], ultimately leading to their increased success and productivity [4, 5, 8]. Over the past decades, reforms have been implemented in the system of higher education through moving from centralized to decentralized (UBM) management [9].

In this regard, the nursing trainers are recommended to pay attention to the necessity of implementation of shared organizational governance by accepting innovations in the healthcare system and applying shared governance in the management style. Shared governance precipitates mission achievements, perspectives, and academic values via making relations and creating common goals and cooperation among all members [2]. Shared governance is a structure that strengthens cooperation, joint decision-making, justice, possession, and responsibility among the stakeholders to support the improvement of quality of the academic learning environment [2, 5, 7]. Despite the similarities observed in the characteristics of shared governance given in various definitions of the term, the concept has no definite and crystal-clear meaning. This is due to the differences in the socio-cultural context of various communities [10].

In Iran, the concept of shared governance is not well known. It is often confused with collaborative management or participatory management. Few studies have been carried out with respect to shared governance from educational organizations’ perspective [2]. No study has been conducted or reported so far in the Iranian educational centers with their different cultural features on shared governance. Therefore, given that an identification of the predisposing factors and antecedents which occur before the incidence of the concept will be very helpful in implementing the shared governance [11], this study explored the antecedents of shared governance in nursing schools of three major medical universities of Tehran, Iran.

## Methods

### Design

This qualitative study conducted in 2016–2017 used conventional content analysis to analyze the narrative data [12].

### Participants

Eleven participants including one dean of nursing school, three managers of departments, and seven faculty members selected respectively from senior and medium managers of three major medical universities of Tehran and three nursing schools affiliated to them participated in this qualitative study. The participants were selected using purposive sampling method with greatest possible variety in gender, age, work experience, and specialty. The inclusion criteria were inclination for voluntary participation, i.e., without any obligation and with their own consent, and at least 5 years of work experience. Six participants were female and five were male. The youngest was 41 and the oldest was 56 years old. The work experience ranged from 6 to 29 years while the managerial experience ranged from 1 to 24 years. All participants except one general practitioner were specialists in management of nursing schools.

### Data collection

The second author of this paper had individual in-depth semi-structured interviews with the participants vis-à-vis during October 2016 to June 2017. All the interviews were carried out in the participants’ office on their request. Nobody was present in the interview room except the second author and the interviewee. Each interview lasted from 50 to 75 min. The interviews started with a general question: “Please explain about your working conditions at this institution”. The interview was co-constructed, i.e., progressed under the influence of the participants’ response, on the basis of the interviewee’s experiences and varied from person to person. The data saturation took place in the eighth interview. To increase data accuracy, two faculty members and one medium level manager were also interviewed which resulted in no new data. No interview was repeated with any participant. The interviews were recorded as voice files and transcribed verbatim.

### Ethical considerations

The required permission was obtained from the Committee of Ethics in Human Research at Shahid Beheshti University of Medical Sciences. The participants took part in the study voluntarily and were assured of the right to leave the study at any stage. The research goals and procedures were explained to the participants and informed written consent was obtained from each of them. Moreover, principles of anonymity and confidentiality of information were observed.

### Data analysis

Conventional content analysis was used to analyze the data [12] . The second author listened to the interviews carefully several times immediately after their completion to arrive at a general understanding of their content. The interviews were transcribed verbatim and then the unit of analysis, meaning (semantic) unit, condensed meaning unit, and primary codes were determined. The subcategories were distilled by cross-comparing of codes and classifying the similar codes together. Next, the similar subcategories were placed into more abstract categories.

### Rigor

Lincoln & Guba criteria were used to ensure of accuracy and consistency of qualitative data [13]. The researchers devoted sufficient time to immerse themselves in the data, survey the subject under study, and interact with the participants to increase data credibility. The codes obtained from the interviews were investigated by two other authors and three independent researchers to reach consensus. Additionally, the validity of the data was reinforced with peer-checks by three participants. To foster data confirmability, the research steps, procedures, methodology, and decisions made during various stages were elucidated in detail. The context of the study and the characteristics of the participants were fully described to enhance judgment of the transferability for the readers.

## Results

Data analysis resulted in 4 categories and 12 subcategories as shown in Table 1.

**Table 1:** The distilled categories and subcategories

Category	Subcategory
The participatory context of higher education institutions	Uniqueness of the schools/higher education centers, uniqueness of faculty members, uniqueness of nursing education
Infrastructural obligations	The facilitative rules of participation, corresponding intraorganizational support, resources suitable for work, learning the teamwork
Coordination with contemporary needs	The need of communities for participation, the variable environment of the organizations
Participation-oriented managers	Managers as symbols of participation, the intrinsic and acquired competencies of the managers, involving faculty members in school/higher education centers management

### Category 1: The participatory context of higher education institutions

The higher education centers are more apt to decentered and participatory management than other organizations due to their knowledge-based nature, professional staff, and experts. This antecedent consists of three subcategories including “uniqueness of schools/higher education centers”, “uniqueness of faculty members”, and “uniqueness of nursing education”.

#### Subcategory 1: Uniqueness of schools/higher education centers

The complex structure and educational and research mission of higher education institutions and the necessity of the presence of dynamism in them have turned these organizations into a susceptible milieu for participation and sharing. A faculty member with 27 years of experience in nursing education put it this way: “We need dynamism in our school; we need to progress all the time. So, every day of our lives should be different from our previous day. Participation in higher education ought to be stronger than that in any other organization”.

A medium level manager with 18-years history of management experience told: “In the academic field, we do not expect hierarchical system at all. We have the Board of Trustees at the University that extends the scope of participation even outside the university.”

#### Subcategory 2: Uniqueness of faculty members

The participants’ belief in the necessity of participation in educational institutions due to their expert and powerful staff led to the formation of this subcategory. A faculty member with 27 years of experience in nursing education asserted: “We may not apply a beurocratic and centered management in schools as is the case in many other organizations because they enjoy knowledgeable and thoughtful personnel each of whom possess a specific type of knowledge and experience”.

A dean of nursing school with 24-years history of management experience told: “Administrators of higher education institutions need to act in a collaborative and participative manner; if they are pretending to participate at meetings, faculty members will notice this demonstration due to their high level of intelligence, and will lose their trust in the manager.”

#### Subcategory 3: Uniqueness of nursing education

The mission of nursing schools in saving human lives confirms the necessity of participation and cooperation among all the members with different specialties at all organizational levels. Another faculty member with 17-years of experience in nursing education postulated: “We deal with human lives in nursing; since the role of the nursing school compared to the role of other parts of the nursing field is like the relation of the brain to the whole body, hence participation should be much stronger in the nursing schools that guide other sections”.

A faculty member with 21 years of experience in nursing education asserted: “Because there are different disciplines in nursing schools, everyone should be involved in decision making. Even the dean of our school who is not expert in some issues, sometimes says to me; I do not know, would you please explain me about this issue?”

### Category 2: Infrastructural obligations

To implement shared governance successfully, there should be not only facilitative rules and regulations of participation and corresponding intra-organizational support, but also suitable work resources for the personnel provided by the managers, and teamwork training must be the priority of all the personnel and managers. This category consists of four subcategories including “facilitative rules of participation”, “corresponding intra-organizational support”, “resources suitable for work”, and “learning the teamwork”.

#### Subcategory 1: Facilitative rules of participation

If the present rules do not induce autocratic management in the managers, are stable and clear, and urge participatory performance of the members, they may create a suitable context for shared governance and validate it.

A dean of nursing school with 24-years management experience told: “Rules and regulations can develop participatory spirit or they may hinder it. Shared governance means that our rules must be based on sharing and participation. I need to be seen as a member in this setting. I wana be seen in rules and structures”.

A faculty member with 27 years of experience in nursing education told: “I saw in our college the rules that addressed the needs of the staff, recognized their habits, and helped them to improve their performance and use all of their capabilities, and these laws would result in mutual respect. So, greater participation was seen in the organization.”

#### Subcategory 2: Corresponding intra-organizational support

The participants’ emphasis on the necessity of support among the members and the importance of support among the managers and members resulted in the extraction of this subcategory. A participant with 1-year managerial experience said in this regard: “The dean of our school always supports us; we as personnel should help and support each other, too”. A faculty member with 21 years of experience in nursing education told: “As the head of a department, I expect my dean to support me and my decision when I take a decision about one of the members of my department.”

#### Subcategory 3: Resources suitable for work

This subcategory designates the importance of provision of required facilities for performing the right work promptly. In the participants’ view, the presence of manpower with participatory spirit suitable with workload and its variety is one of the most important prerequisites of correct participation. Another participant with 29 years of work experience put it in this way: “Yesterday, there was a meeting for choosing the top professors. I was very inclined to take part in the session as it was highly valuable for me; yet, I couldn’t do so due to numerous duties and responsibilities and I could not attend the school promptly due to the practical training I had”.

A medium level manager with 9-years history of management experience told: “The sufficient number of faculty members is important for participation in the affairs of the organization. However, sometimes, despite enough faculty members and even the presence of collaborative executive managers, there is no effective participation in the organization. Maybe, one of the reasons of that is the presence of employees who do not understand and do not believe in the partnership. In my opinion, we should pay attention to the partnership characteristics of the faculty members in their recruitment.”

#### Subcategory 4: Learning the team work

The participants declared that lack of training or insufficient instruction during infancy or even in organization serves as one of the most important barriers in the way of participatory completion of affairs; therefore, they emphasized the acquisition of communication and participatory skills which led to the emergence of this subcategory. A faculty member with 9 years of experience in nursing education said: “I see, for example, in my work environment that some individuals like to participate; yet, they don’t enter the scene when they don’t have the required knowledge and skill for team work. Team work, team assessment, the use of opinions, their summarizing and prioritization in participation all need skills that ought to be taught”. Another faculty member with 29 years of experience in nursing education told: “When we are forming teams for teamwork, we must be aware of our duties in the team, and we must also know how to communicate with others, superiors, and subordinates.”

### Category 3: Coordination with contemporary requirements

The participation principle or shared governance has been introduced as a valuable issue for communities and organizations at the international level. In addition, people, especially the youth in Iran, have gained an increased awareness and inclination for intervention in their own affairs at the family and country level [14]. Hence, the need for participation as one of the contemporary requirements in Iran is felt more than ever to pave the way for the acceptance of shared governance in higher education institutions. This category consists of two subcategories including “the need of communities for participation”, and “the variable environment of organizations.”

#### Subcategory 1: The need of communities to participation

Following the increased knowledge and awareness of the youth in Iran resulting from mass media and higher education and increased attention of families to their children, the demand for participation is increasing constantly. A medium level manager with 9 years history of management experience told: “Nowadays, the inclination for participation is not just related to the university. Even ourselves we see that primary or high school students are very interested in participation”.

Another faculty member with 27 years of experience in nursing education told: “Increasing awareness of the people in different ways, such as academic and media, all made people more active and caused them to be more involved”.

#### Subcategory 2: The variable environment of organizations

The increasing wants and wishes of the Iranian society and the organizations supervising the educational institutions in Iran to update and enhance output serve as a stimulator that precipitates the implementation of shared governance and managers’ use of expert opinions of the staff. Another faculty member with 12 years of experience in nursing education put it this way: “Seeing that the organizations are constantly changing, today, as a manager, I expect to be able to lead my organization well, and bring about much achievement in my organization. All these require sharing and participation”.

A medium level manager with 10 years history of management experience told: “Nowadays, due to rapid changes in all aspects of society and technology, all organizations, not just the higher education institutions, have been in a challenging situation, which has led to the need for managers to engage more and more specialist staff to overcome these challenges”.

### Category 4: Participation-oriented managers

The last antecedent of the present study emphasizes the importance of beliefs and behaviors demanding the participation of managers of higher education institutions in successful implementation of shared governance and includes three subcategories: “managers as symbol of participation”, “the intrinsic and acquired managerial competencies of the managers”, and “involving faculty members in school/higher education centers management”.

#### Subcategory 1: Managers as symbol of participation

The senior managers are the model of participatory performance in schools and can play a highly effective role in creating the participatory culture even if the school rules are non-participatory. Another faculty member with 17 years of experience in nursing education postulated in this regard: “With the experience that I have been teaching and managing over the years, to implement the shared governance, the key authorities of the organization such as the dean and assistant directors ought to believe in the participation principle and implement it”.

Another faculty member with 6 years of experience in nursing education said: “The dean of our school has such a great deal of leadership power that he creates a sense of participation in people who do not have any participation background; when they enter our school, they seem to be another person and have motivation for participation”.

#### Subcategory 2: The intrinsic and acquired managerial competencies of the managers

The participants believed that for successful participation, only individuals who possess a series of inherent characteristics like talent, creativity, risk-taking, leadership, and the ability for adjusting rules in line with participation and also enjoy suitable scientific history with a high position and managerial knowledge and train their subordinates should be appointed to managerial posts .

A senior manager with 18 years history of management experience said: “The position of school dean requires an academic person. They should possess some personality and moral characteristics, some abilities and capacities with suitable scientific and managerial history”.

Another faculty member with 17 years of experience in nursing education said: “I saw some managers who have a lot of understanding of participation, emotional intelligence, understanding the interests of the faculty members, and making them part of the partnership.”

#### Subcategory 3: Involving faculty members in school/higher education centers management

Only managers are successful in implementing shared governance that let the faculty members perform their educational and research duties independently and involve them in managerial affairs as well. Another faculty member with 26 years of experience in nursing education said: “Since faculty members are thoughtful individuals, they have much experience and knowledge; this knowledge and experience should be used in managing the school”.

Another faculty member with 6 years of experience in nursing education said: “The dean and vice-dean of our school are closely collaborating with others especially the head of departments for school managing. The views and positions of the head of the department are important to her (dean of school); she said at the meetings the head of department is my representative”.

## Discussion

This study aimed at determining the antecedents of shared governance. The findings obtained from analyzing the culled data resulted in the emergence of 4 categories and 12 subcategories. The first category distilled was “the participatory context of higher education institutions” with three subcategories including “uniqueness of schools”, “uniqueness of faculty members”, and “uniqueness of nursing education”. The participants emphasized the uniqueness of schools. Similar studies also reported that universities require shared governance due to the fact that they are a form of professional organizations with educational, research, and service-provision goals which have complex duties working in a competitive variable environment [1, 8, 15]. Moreover, academic shared governance is expressed as an essential component of the influential statement of American Association of University Professors in 1996 and is necessary for reinforcement and success of higher education centers as it is in line with the concepts of adult education [16]. Furthermore, uniqueness of faculty members is in line with studies that render shared governance as necessary for higher education institutions due to the presence of expert and professional personnel and for empowerment of faculty members in managing universities [1, 8, 15] . Moreover, the participants believed in the uniqueness of nursing education so that similar studies also referred to the differences in the type of governance and organizational structure and basic columns of decision-making from one university to another [17] and lack of obligation of institutions for having a specific recommended model of governance of faculty members [8]. Additionally, shared governance has been introduced as a method of coping diversity among faculty members and personnel of nursing schools and improving just and fair decision-making. Also, concerns on improving the work setting are increasing in nursing schools owing to the existence of a great number of open academic nurse educator positions [2]. In this study, the participants noted the necessity of the presence of infrastructures as a major category. For instance, the facilitative role of rules introduced by the participants as an infrastructure is consistent with the studies that emphasized the extensive implementation of shared governance in the universities and schools of US following the joint statement in 1996 by AAUP, ACE, and AGB. This statement entails the two principles of participation of all institution members and identifying the weight of each vote on the basis of the rate of responsibility of each member in specific subjects and supports Ramo’s shared governance indicators [1, 7, 8, 17]. Regarding corresponding intra-organizational support, similar studies have also emphasized the necessity of support and respect of faculty members towards each other in fundamental and governance responsibilities [1] and also the managers’ support for institution’s strategies [8] and development of governance skills in faculty members [17]. Emphasis has also been put on the necessity of understanding the importance of the support for university president, faculty members, and university students by governing board members [7, 18] with the difference that there is no governing board at the governmental schools in Iran. The participants further mentioned suitable resources of work as essential for shared performance of affairs. This is consistent with studies that render the maintenance of acceptable workload for faculty members [17], the presence of sufficient budget, facilities, and manpower [19], and reducing part-time faculty members as necessary for successful implementation of shared governance [10]. Learning to work as a team was introduced as the last infrastructure by the participants. This is in line with similar studies that mentioned education as a vital issue for shared governance emphasizing the obligation for the presence of current prompt strategies in the organization to reflect experiences and learning from others’ experiences to make participation in decision-makings efficacious [8, 16]. The category “coordination with contemporary needs” consisted of two subcategories of “the need of communities for participation” and “variable organizational environment”. Other studies have also emphasized the contemporary changes which have the potential to modify the decision-making model in educational institutions and emphasize the necessity of shared governance and its adjustment. These changes include issues like the relations between state governments and educational institutions, reduced public support, pressures on the universities to interact with rivals and globalization, and also changing the manpower from full-time to part-time [1, 10]. The participants’ focus on the importance of the role of managers in participation led to the formation of the category “participation-oriented managers”. The subcategory “managers as symbol of participation” is in line with studies that emphasize the role of higher levels of institutional management in creating and reinforcing a cooperative culture of participation and the successful implementation of shared governance [8, 16, 17]. These studies mention the shaping of institutional culture as the most important function of the institute’s leader [7, 8]. Moreover, the participants of the study gave special weight to the intrinsic and acquired competencies of the managers. Similar studies also emphasized the need for features such as leadership and the use of innovative managerial models by managers and the necessity of the presence of strong selection systems for choosing qualified managers [7]. Due to difficulties in university governance, they refer to reasonable and logical governance as an acquired issue and ask managers to learn it [16]. Involving faculty members in school management was also strongly noticed by the participants. These findings are consistent with studies that give the most important role to the faculty members in successful implementation of shared governance [4, 8, 10, 16, 20]. They emphasize the fulfillment of primary duties of faculty members in shared governance like affairs related to education and research and issues related to the students’ lives dealing with education and research [1, 4, 7, 10, 17, 21]. They also emphasize faculty members’ involvement in governing affairs such as planning, budgeting, and relations with extra organizational centers by managers [1, 2, 17, 21]. The study is not without limitations. This qualitative study was conducted on nursing schools, so, the acquisition and use of the experiences of experts in other schools are not possible in this study jeopardizing the external validity of the results; hence, our findings cannot be generalized to other or future situations. Ultimately, it should be pointed out that this study was carried out only in Tehran nursing schools. It is recommended that future research be directed at other nursing schools in Iran and other countries. Our findings may aid the nursing school managers in making preparations to implement the shared governance. It is also recommended that our results be used by managers and scholars to carry out larger scale research on assessing the possibility of implementing shared governance in their own context.

## Conclusion

Our findings demonstrated that factors such as the suitable environment of nursing schools and the need of the Iranian community for participation and intervention in their own fate and the wants of the surrounding environment for increasing the outcome of educational institutions can play a significant role in implementing shared governance in such institutions. Moreover, the results indicated that there is the need for infrastructural obligations like facilitative rules of participation, manpower, and suitable facilities for performing affairs to implement shared governance successfully. Consequently, it appears that some measures ought to be taken at the level of Ministry of Health, Treatment, and Medical Education and at universities. It is also a good idea to select the personnel and especially the managers features such as participatory personality. Finally, the managers are advised to acquire knowledge on shared governance and spread it in their organization, create a supportive environment, and adjust the local rules of their institution to facilitate participation.

## Implications for nursing management

The application of shared governance in all educational institutions, especially nursing schools, which deal with human lives, demands an appropriate context. The selection of competent and participation-oriented managers is one of the most important predisposing factors in this study. In the participants’ view, this factor can strongly help the successful implementation of shared governance even in the absence of other predisposing parameters. Hence, it seems that even in institutions where all activities are done in a hierarchal system under the influence of the centered power of the senior manager, the shared governance may be applied. The higher-level managers of educational institutions can not only provide the infrastructural obligations for performing participatory affairs, but also empower themselves, lower level managers, and their staff in communication and participation skills and serve as the symbol of participatory model in the leadership position. Ultimately, the selection and appointment of the managers of institutions can be accomplished based on intrinsic and acquired characteristics appropriate for participatory performance and team work.

## Abbreviations

AAUP: American Association of University Professors
ABG: American Governing Boards
ACE: American Council of Education
